# Skeletal Muscle Proteomic Profile Revealed Gender-Related Metabolic Responses in a Diet-Induced Obesity Animal Model

**DOI:** 10.3390/ijms22094680

**Published:** 2021-04-28

**Authors:** Manuela Moriggi, Sara Belloli, Pietro Barbacini, Valentina Murtaj, Enrica Torretta, Linda Chaabane, Tamara Canu, Silvia Penati, Maria Luisa Malosio, Antonio Esposito, Cecilia Gelfi, Rosa Maria Moresco, Daniele Capitanio

**Affiliations:** 1 Gastroenterology and Digestive Endoscopy Unit, IRCCS Policlinico San Donato, 20097 San Donato Milanese, Italy; manuela.moriggi@grupposandonato.it; 2 Institute of Molecular Bioimaging and Physiology, CNR, 20090 Segrate, Italy; sara.belloli@ibfm.cnr.it (S.B.); rosa.moresco@unimib.it (R.M.M.); 3 Department of Nuclear Medicine, IRCCS San Raffaele Scientific Institute, 20132 Milan, Italy; murtaj.valentina@hsr.it; 4 Department of Biomedical Sciences for Health, University of Milan, 20090 Segrate, Italy; pietro.barbacini@unimi.it (P.B.); cecilia.gelfi@unimi.it (C.G.); 5 PhD Program in Neuroscience, School of Medicine and Surgery, University of Milano-Bicocca, 20900 Monza, Italy; 6 IRCCS Istituto Ortopedico Galeazzi, 20161 Milan, Italy; enrica.torretta@grupposandonato.it; 7 Experimental Imaging Center, Preclinical Imaging Facility, IRCCS San Raffaele Scientific Institute, 20132 Milan, Italy; chaabane.linda@hsr.it (L.C.); canu.tamara@hsr.it (T.C.); esposito.antonio@unisr.it (A.E.); 8 Laboratory of Pharmacology and Brain Pathology, Neuro Center, IRCCS Humanitas Research Hospital, 20089 Rozzano, Italy; silvia.penati@hunimed.eu (S.P.); maria_luisa.malosio@humanitasresearch.it (M.L.M.); 9 Institute of Neuroscience, Humanitas Mirasole S.p.A, 20089 Rozzano, Italy; 10 Experimental Imaging Center, Radiology Department, IRCCS San Raffaele Scientific Institute, School of Medicine, Vita-Salute San Raffaele University, 20132 Milan, Italy; 11 Department of Medicine and Surgery, University of Milano-Bicocca, 20900 Monza, Italy

**Keywords:** insulin resistance, obesity, proteomics, sarcopenia, skeletal muscle

## Abstract

Obesity is a chronic, complex pathology associated with a risk of developing secondary pathologies, including cardiovascular diseases, cancer, type 2 diabetes (T2DM) and musculoskeletal disorders. Since skeletal muscle accounts for more than 70% of total glucose disposal, metabolic alterations are strictly associated with the onset of insulin resistance and T2DM. The present study relies on the proteomic analysis of gastrocnemius muscle from 15 male and 15 female C56BL/J mice fed for 14 weeks with standard, 45% or 60% high-fat diets (HFD) adopting a label-free LC–MS/MS approach followed by bioinformatic pathway analysis. Results indicate changes in males due to HFD, with increased muscular stiffness (Col1a1, Col1a2, Actb), fiber-type switch from slow/oxidative to fast/glycolytic (decreased Myh7, Myl2, Myl3 and increased Myh2, Mylpf, Mybpc2, Myl1), increased oxidative stress and mitochondrial dysfunction (decreased respiratory chain complex I and V and increased complex III subunits). At variance, females show few alterations and activation of compensatory mechanisms to counteract the increase of fatty acids. Bioinformatics analysis allows identifying upstream molecules involved in regulating pathways identified at variance in our analysis (Ppargc1a, Pparg, Cpt1b, Clpp, Tp53, Kdm5a, Hif1a). These findings underline the presence of a gender-specific response to be considered when approaching obesity and related comorbidities.

## 1. Introduction

Recently, obesity incidence has dramatically increased, reaching pandemic proportions with a rising trend until 2030 [1]. Obesity is due to an abnormal fat accumulation resulting from a long-term energy imbalance between caloric intake and energy expenditure [2,3]. It is a chronic and complex pathological condition associated with cardiovascular diseases, cancer, type 2 diabetes mellitus and musculoskeletal disorders, like sarcopenia [4] and tendinopathies. In obese subjects, tendons frequently undergo functional impairment and degeneration caused by the increased load and the presence of systemic dysmetabolic factors, as non-load-bearing tendons resulted affected, especially in male subjects [5,6]. Multiple risk factors are directly or indirectly associated with obesity; among them, environmental factors [7,8], genetics [9], gender [10], aging [11], gut microbiota [12], and diets [13,14] are prominent. For such a reason, in the scientific community, the association between obesity and comorbidities has been widely studied, highlighting insulin resistance (defined as reducing glucose uptake in response to the effects of insulin) as the main link between obesity and the onset of pathological processes. Among the mechanisms leading to systemic insulin resistance in obese subjects, one is adipose tissue failure due to high free fatty acids (FFAs) levels. Adipose tissue is the body compartment where excess FFAs are stored. When the level of FFAs exceeds the storage capacity of adipocytes, they compensate by increasing their number (hyperplasia) or their size (hypertrophy). However, over a certain level of FFAs, the compensatory mechanism of adipocytes increment and remodeling fails, leading to accumulation of FFAs in non-adipose tissues, such as liver and skeletal muscles, causing lipotoxicity [15,16]. The toxic lipids then dysregulate mitochondria, endoplasmic reticulum and lysosomes. Dysfunctional organelles promote the release of FFAs and their intermediates (e.g., diacylglycerol, linoleic, phosphatidic and lysophosphatidic acids and ceramides), proinflammatory molecules and ROS. The increase of these molecules in the bloodstream [17,18] can develop a low-grade systemic inflammation that determines the onset of insulin resistance [19].

Among tissues affected by metabolic alterations, skeletal muscle plays a pivotal role [20]. It accounts for up to 40% of the total body mass, it is involved in a plethora of biological functions, including movement regulation and heat homeostasis control, and it requires a high energy supply being responsible for more than 70% of glucose uptake [15]. Muscle metabolic alterations are responsible for the onset and worsening of insulin resistance, leading to type 2 diabetes (T2DM) and sarcopenia. The combination of skeletal muscle loss coupled to fat gain, defined as sarcopenic obesity, has been associated with a higher risk of metabolic syndrome, cardiovascular and liver disease, frailty, mobility impairment and mortality [21,22]. For this reason, the study of muscle metabolic alterations represents an important target allowing identifying altered pathways that could be pharmacologically treated or could lead to the discovery of novel biomarkers [23,24,25].

The high metabolic rate of skeletal muscle is highlighted by its complex proteome profile characterized by a dynamic range of six orders of magnitude in protein concentrations, a trait that makes the experimental study of this biological sample challenging [26]. During the last decades, significant advances in the study of skeletal muscle proteome under different biological conditions have been made thanks to the introduction of high-throughput technologies, like mass spectrometry (MS) coupled with very efficient separative methods (e.g., two-dimensional gel electrophoresis (2-DE) and high-performance liquid chromatography (HPLC)) and recently by applying the bottom-up approach coupled with a new generation of high-resolution mass spectrometers [27,28,29,30]. The latter allowed an indirect quantitative measurement by analyzing peptides resulting from the proteolytic digestion of intact proteins.

This study aims to characterize the proteomic profile of skeletal muscles of diet-induced obesity (DIO) animal models fed with high-fat diets at the different fat content. Since obesity and related comorbidities are influenced by several factors, including gender, males and females have been studied separately and compared. Alterations of both contractile and metabolic proteins have been considered to get insights into this complex pathological context to recognize potential new therapeutic targets and biomarkers. Given the pandemic proportion that obesity has reached, the results of this study can impact health, social and economic levels.

## 2. Results

### 2.1. High-Fat Regimen Effects and Gender Response

**Figure 1:**
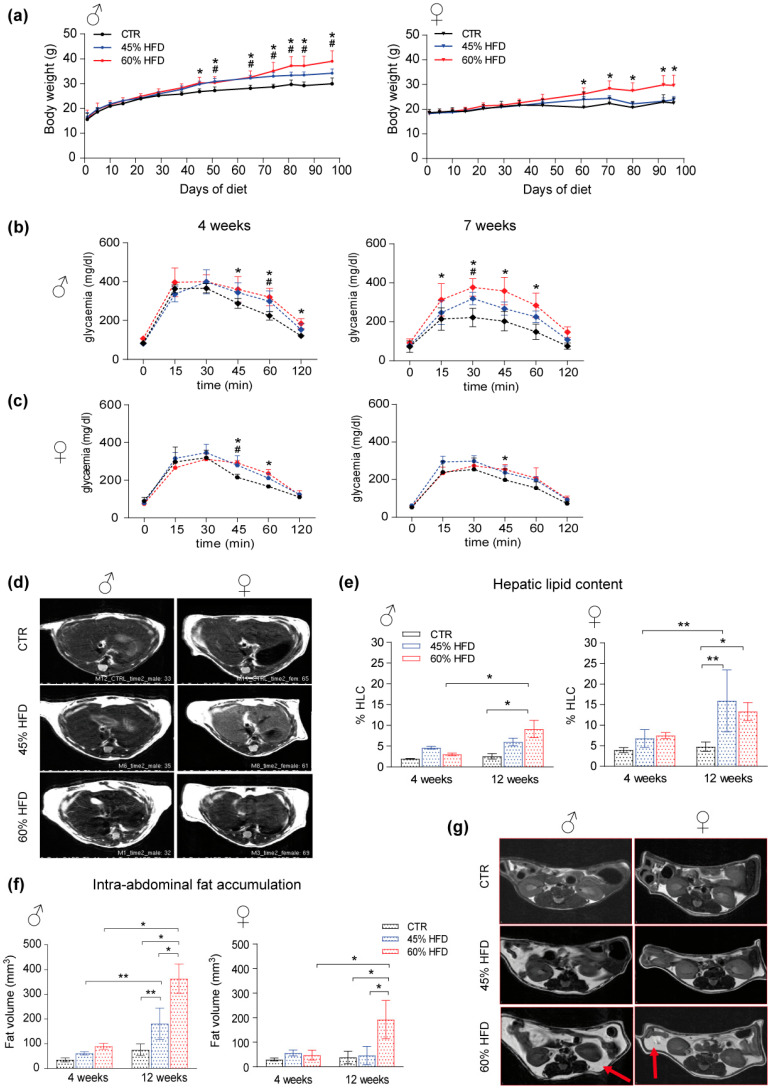
High-fat animal model characterization. (**a**) male (♂, left) and female (♀, right) mice body weight increase (* significant variation in 60% HFD vs. CTR, # significant variation in 45% HFD vs. CTR). Data were analyzed using two-way ANOVA for diet and time point as variables, followed by Tukey’s post hoc test for multiple comparisons (n = 4, *p*-value < 0.05). (**b**) Longitudinal glucose tolerance test in male and (**c**) female HFD mice at 4 and 7 weeks of diet (* significant variation in 60% HFD vs. CTR, # significant variation in 45% HFD vs. CTR). Data were analyzed using two-way ANOVA in which within each row, each column mean was compared, followed by Tukey’s post hoc test for multiple comparisons (n = 4, *p*-value < 0.05). (**d**) T2-MRI images of male (left) and female liver (liver) section per group of study (CTR, 45% HFD and 60% HFD) at 12 weeks of diet. Non-fat suppression images in which whiter liver reveals higher lipid content. (**e**) % Hepatic lipid content (% HLC) spectroscopy evaluation on male (left) and female (right) liver section at 4 and 12 weeks of diet (* significant variation *p*-value < 0.05, ** significant variation *p*-value < 0.01). (**f**) Fat volume longitudinal quantification in male (left) and female (right) mice at 4 and 12 weeks of diet. Data were analyzed using two-way ANOVA for diet and time point as variables, followed by Tukey’s post hoc test for multiple comparisons. All data were expressed as mean ± SD (* significant variation *p*-value < 0.05, ** significant variation *p*-value < 0.01). (**g**) T2-MRI representation of male (left) and female (right) intra-abdominal fat accumulation per group of diet (CTR, 45% HFD and 60% HFD) at 12 weeks of diet; red arrows indicate fat tissue accumulation.

Animals fed for 14 weeks with two high-fat diets (HFD) regimens (45 kcal% and 60 kcal% fat content diets; 45% HFD and 60% HFD, respectively) showed increased body weight compared to a control group (CTR) fed with a standard (10 kcal% fat content) diet (Figure 1a). This result was more evident for male mice in which both types of diet caused a significant increment in body weight that started from 7 weeks and was maintained until the end of the experiment (Figure 1a, left panel). Conversely, female mice exhibited less increase in body weight, and only the 60% HFD showed significant results compared to the control group (Figure 1a, right panel). Glucose tolerance test (GTT), performed to assess glucose disposal by peripheral organs, indicative of insulin resistance, revealed higher levels of glycemia in both sexes, particularly evident in 60% HFD-fed males. In males, glucose intolerance occurred earlier, starting already after 4 weeks of diet, whereas, in females, it appeared at 7 weeks, although the effect was lower compared to males (Figure 1b,c).

The effect of HFD on hepatic lipid content was evaluated using proton nuclear magnetic resonance (1H-NMR) spectroscopy at 4 and 12 weeks of diet (Figure 1d). An increase in total hepatic lipid content (% HLC) was detected only after 12 weeks in both male and female mice (Figure 1d). HFD male mice exhibit % HLC significantly increased after 12 weeks compared to 4 weeks and controls. The increase was limited to the 60% HFD group (Figure 1e, left). Conversely, in female mice, the response was higher after the 45% HFD regimen (Figure 1e, right). % HLC at 12 weeks was significantly higher compared to 4 weeks and controls. Moreover, female lipid content at 12 weeks for 60% HFD was also significantly higher than in controls. Intra-abdominal fat accumulation, representing another hallmark of metabolic disorders, was measured with transverse relaxation time–magnetic resonance imaging (T2-MRI). The analysis revealed increased fat volume at 12 weeks, higher in male than in female HFD-fed mice (Figure 1f,g). For both sexes, the increase was significant after 60% HFD and for males also after 45% HFD.

### 2.2. Proteomic Profiles of Skeletal Muscle in High-Fat Diet

**Figure 2:**
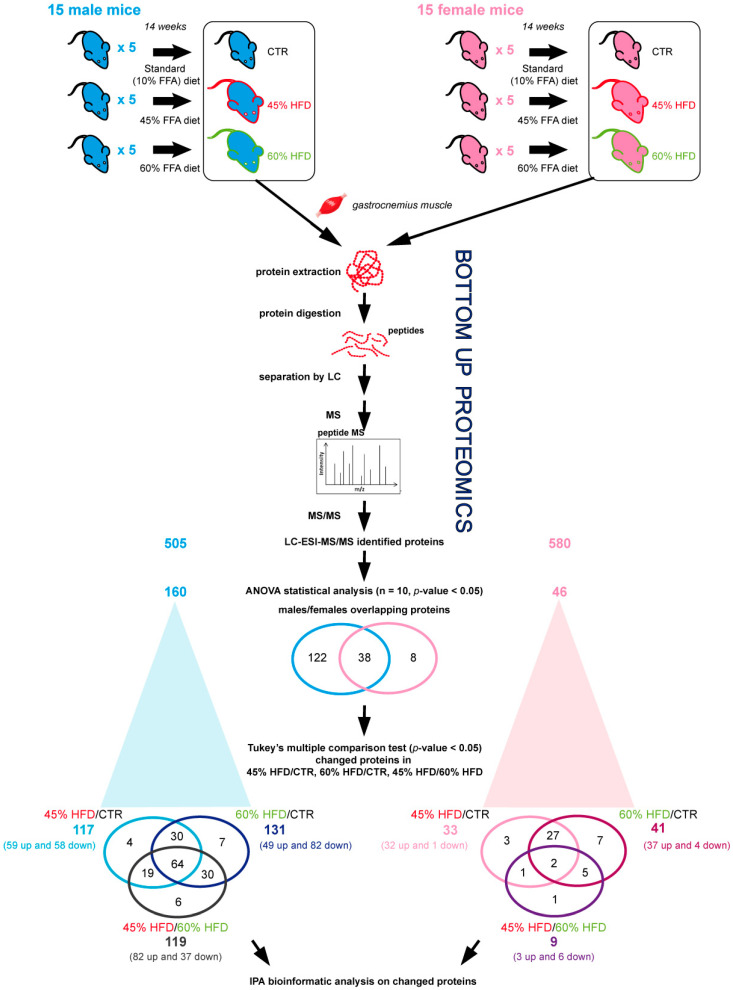
Label-free LC–ESI–MS/MS experimental design and main results. Protein extracts from 15 male and 15 female mice fed for 14 weeks with a standard diet (10 kcal% fat content; CTR) and two high-fat diets (HFD) regimens (45 kcal% and 60 kcal% fat content diets; 45% HFD and 60% HFD, respectively). For both genders, 5 samples for each condition were collected and analyzed in duplicate using label-free LC–ESI–MS/MS protocol. Three different comparisons were performed: 45% HFD vs. CTR, 60% HFD vs. CTR and 45% HFD vs. 60% HFD. The ANOVA test (n = 10, *p*-value > 0.05) showed 160 and 46 proteins differentially regulated in males and females, respectively. Of them, 38 proteins were in common between males and females. Specifically, comparing 45% HFD vs. CTR, 117 and 33 differentially abundant proteins were found, in males and females, respectively (Tukey’s multiple comparison test, *p*-value < 0.05). In contrast, the same analysis highlighted 131 and 41 differentially abundant proteins in 60% HFD vs. CTR males and females, and 119 and 9 differentially abundant proteins in 45% HFD vs. 60% HFD males and females, as detailed in Supplementary Tables S1 and S2.

Gastrocnemius muscle protein extracts were analyzed by liquid chromatography coupled to electrospray tandem mass spectrometry (LC–ESI–MS/MS) with label-free quantification to evaluate changes in protein abundance among CTR, 45% HFD, and 60% HFD-fed male and female mice for 14 weeks (Figure 2). Overall, ANOVA test (n = 10, *p*-value < 0.05) results revealed 160 changed out of 505 identified proteins in males and 46 changed out of 580 identified proteins in female mice. Tukey’s multiple comparison post hoc test showed that, compared to CTR, 117 out of 160 changed proteins were differently expressed (*p*-value < 0.05) in 45% HFD and 131 in 60% HFD male mice, whereas 119 were changed in 45% vs. 60% HFD. In females, 33 out of 46 changed proteins were differentially expressed in 45% HFD and 41 in 60% HFD than CTR. Nine proteins were changed in 45% vs. 60% HFD. Identification data for changed proteins are shown in Supplementary Tables S1 and S2 for male and female mice, respectively.

#### 2.2.1. Structural Proteins

**Figure 3:**
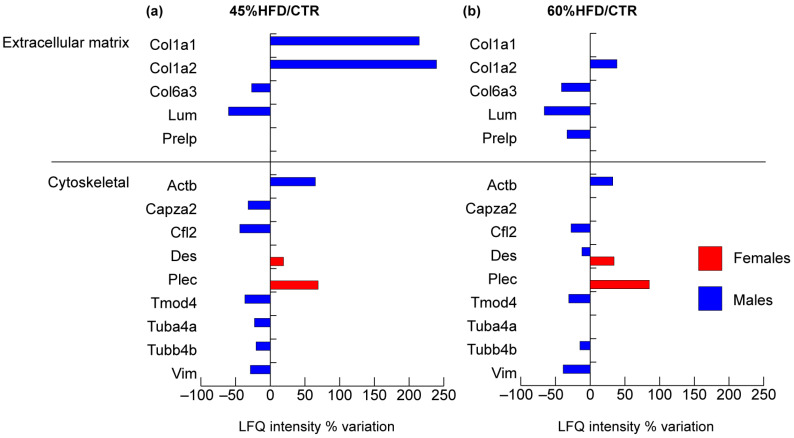
Proteomic analysis of structural proteins. Histograms of differentially expressed proteins (Label-free quantification, LFQ, intensity% variation, ANOVA and Tukey’s test, n = 10, *p*-value < 0.05) in (**a**) 45% HFD vs. standard diet (CTR) female (red bars) and male (blue bars) mice; (**b**) 60% HFD vs. CTR female and male mice.

Considering structural proteins of muscle tissue (Figure 3), the number of extracellular matrix and cytoskeleton proteins identified and changed was higher in male compared to female mice.

Extracellular matrix proteins collagen alpha 1 and 2 (Col1a1 and Col1a2) were increased in 45% HFD, while collagen 6 alpha chain (Col6a3) and lumican (Lum) showed a decrement in both 45% and 60% HFD compared to controls. Prolargin (Prelp) decreased in 60% HFD only.

Only two cytoskeletal proteins were increased in females, in both 45% and 60% HFD compared to controls: desmin (Des) and plectin (Plec); notably, desmin was also changed in 60% HFD males, but with an opposite trend. In males, the following structural proteins were decreased in both 45% and 60% HFD compared to controls: cofilin-2 (Cfl2), tropomodulin-4 (Tmod4), tubulin beta-4B chain (Tubb4b) and vimentin (Vim). Cytoplasmic beta-actin (Actb) increased in all comparisons. In contrast, F-actin-capping protein subunit alpha-2 (Capza2) and tubulin alpha-4A chain (Tuba4a) decreased only in 45% HFD vs. control group.

#### 2.2.2. Contractile Proteins

**Figure 4:**
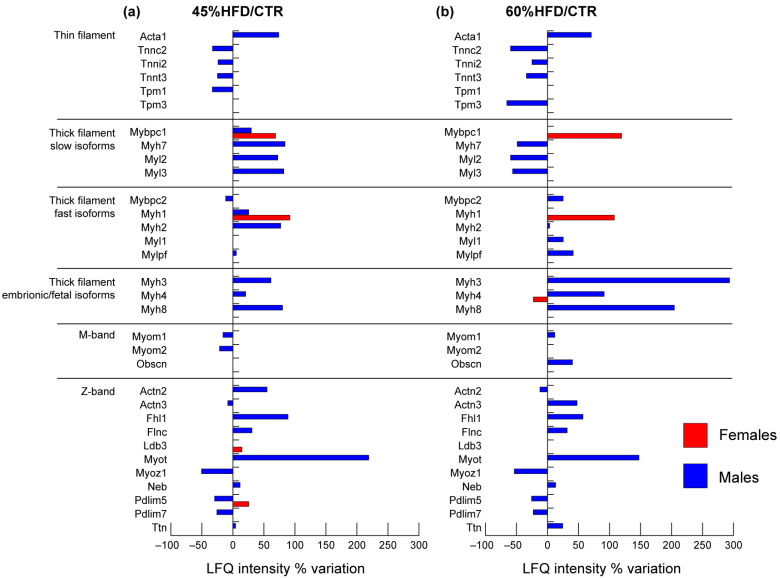
Proteomic analysis of contractile proteins. Histograms of differentially expressed proteins (LFQ intensity% variation, ANOVA and Tukey’s test, n = 10, *p*-value < 0.05) in (**a**) 45% HFD vs. standard diet (CTR) female (red bars) and male (blue bars) mice; (**b**) 60% HFD vs. CTR female and male mice.

In 45% and 60% HFD vs. CTR male mice, troponins from thin filaments (Tnnc2, Tnni2, Tnnt3) decreased, whereas the skeletal muscle isoform of actin alpha (Acta1) increased. Tropomyosin 1 (Tpm1) decreased in 45% HFD, whereas tropomyosin 3 (Tpm3) decreased in 60% HFD vs. CTR (Figure 4).

In males, slow-type thick filament’s proteins increased in 45% HFD and decreased or were unchanged in 60% HFD compared to CTR: myosin 7 (Myh7), myosin regulatory light chain 2 (Myl2), myosin light chain 3 (Myl3) and myosin-binding protein C (Mybpc1). On the other hand, fast-type proteins myosin 1 (Myh1), myosin 2 (Myh2) and myosin regulatory light chain 2 (Mylpf) were increased, while myosin-binding protein C (Mybpc2) decreased in 45% HFD compared to controls. In 60% HFD vs. CTR, all these contractile proteins increased except for Myh1, which was unchanged. Myosin light chain 1/3 (Myl1) increased in 60% HFD vs. CTR only. Perinatal myosin 8 (Myh8), embryonic myosin 3 (Myh3) and fetal myosin 4 (Myh4) were increased both in 45% HFD and 60% HFD compared to control. In females, only three proteins of the thick filament were changed in abundance: Myh1 and Mybpc1 increased in both 45% and 60% HFD vs. CTR, whereas Myh4 decreased in 60% HFD vs. CTR.

In males, proteins of the M-band were decreased in 45% HFD vs. CTR: myomesin-1 (Myom1) and myomesin-2 (Myom2). The former reverted its trend, whereas the latter was unchanged in 60% HFD compared to CTR. Obscurin (Obscn) showed upregulation in 60% HFD vs. CTR only.

In the Z-band of male mice, the following proteins changed in both 45% and 60% HFD vs. CTR: four and a half LIM domains protein 1 (Fhl1), filamin C (Flnc), myotilin (Myot), nebulin (Neb) and titin (Ttn) increased, while myozenin 1 (Myoz1) and PDZ and LIM domain proteins 5 and 7 (Pdlim5, Pdlim7) decreased. In 45% HFD vs. CTR, alpha-actinin 2 (Actn2) increased, whereas alpha-actinin 3 (Actn3) decreased; these proteins were also changed in 60% HFD males with an opposite trend. In females, only the Pdlim5 and LIM domain-binding protein 3 (Ldb3) were increased in 45% HFD.

#### 2.2.3. Glucose and Glycogen Metabolism

**Figure 5:**
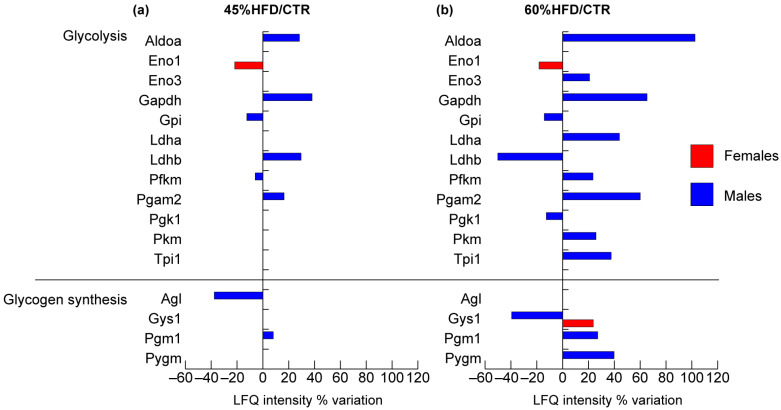
Proteomic analysis of glucose/glycogen metabolism proteins. Histograms of differentially expressed proteins (LFQ intensity% variation, ANOVA and Tukey’s test, n = 10, *p*-value < 0.05) in (**a**) 45% HFD vs. standard diet (CTR) female (red bars) and male (blue bars) mice; (**b**) 60% HFD vs. CTR female and male mice.

Several glycolytic proteins were identified in males compared to females in both the HFDs (Figure 5), as previously observed for structural and contractile proteins. In males, aldolase A (Aldoa), glyceraldehyde-3-phosphate dehydrogenase (Gapdh), and phosphoglycerate mutase 2 (Pgam2) increased in both HFDs models compared to controls. Glucose-6-phosphate isomerase (Gpi), ATP-dependent 6-phosphofructokinase (Pfkm), were decreased in 45% HFD vs. CTR, whereas they were, respectively, decreased and increased in 60% HFD compared to controls. Pyruvate kinase (Pkm) and triosephosphate isomerase (Tpi1) increased, and phosphoglycerate kinase 1 (Pgk1) decreased in 60% HFD vs. CTR. The enolase complex was at variance in male and female samples. In males, enolase 3 (Eno3) increased in 60% HFD compared to the standard diet, whereas in females, enolase 1 (Eno1) decreased in both 60% and 45% HFD compared to controls. Finally, lactate dehydrogenase B (Ldhb) increased in 45% HFD and decreased in 60% HFD vs. CTR, whereas lactate dehydrogenase A (Ldha) increased in 60% HFD.

The glycogen biosynthetic pathway was altered in both genders since glycogen synthase 1 (Gys1) increased in females and decreased in males in 60% HFD vs. CTR. In males, phosphoglucomutase 1 (Pgm1) was increased in both HFDs models compared to controls, whereas glycogen debranching enzyme (Agl) was decreased in 45% HFD vs. CTR and glycogen phosphorylase (Pygm) was increased in 60% HFD vs. CTR.

#### 2.2.4. TCA Cycle and Fatty Acids Metabolism

**Figure 6:**
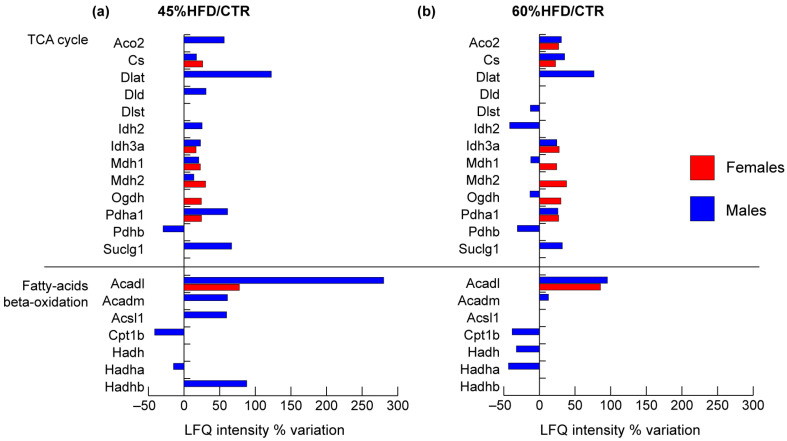
Proteomic analysis of TCA cycle and fatty acids metabolism proteins. Histograms of differentially expressed proteins (LFQ intensity% variation, ANOVA and Tukey’s test, n = 10, *p*-value < 0.05) in (**a**) 45% HFD vs. standard diet (CTR) female (red bars) and male (blue bars) mice; (**b**) 60% HFD vs. CTR female and male mice.

Considering the enzymes of the TCA cycle, it was possible to notice a general increment in both males and females (Figure 6). In males, the following proteins increased in both 45% and 60% HFD vs. CTR: aconitate hydratase (Aco2), citrate synthase (Cs), dihydrolipoyllysine-residue acetyltransferase component of pyruvate dehydrogenase complex (Dlat), isocitrate dehydrogenase subunit alpha (Idh3a), Pyruvate dehydrogenase E1 component subunit alpha (Pdha1) and succinate-CoA ligase subunit alpha (Suclg1), whereas, pyruvate dehydrogenase E1 component subunit beta (Pdhb) decreased in both conditions. Isocitrate dehydrogenase (Idh2) and cytoplasmic malate dehydrogenase (Mdh1) increased in 45% HFD vs. CTR and decreased in 60% HFD vs. CTR. Dihydrolipoyllysine-residue succinyltransferase component of 2-oxoglutarate dehydrogenase (Dlst) and 2-oxoglutarate dehydrogenase (Ogdh) decreased only in 60% HFD vs. CTR, whereas mitochondrial malate dehydrogenase (Mdh2) increased only in 45% HFD vs. CTR. In females, the following proteins increased in both 45% and 60% HFD vs. CTR: Cs, Idh3a, Mdh1, Mdh2, Ogdh and Pdha1, while Aco2 increased only in 45% HFD vs. CTR.

Among enzymes belonging to beta-oxidation, in 45% HFD vs. CTR males, the trifunctional enzyme subunit alpha (Hadha) and carnitine O-palmitoyltransferase 1 (Cpt1b) decreased. In contrast, long-chain-fatty-acid-CoA ligase 1 (Acsl1), trifunctional enzyme subunit beta (Hadhb), long-chain specific acyl-CoA dehydrogenase (Acadl) and medium-chain specific acyl-CoA dehydrogenase (Acadm) were increased. In 60% HFD, Acadl and Acadm increased, whereas Cpt1b, hydroxyacyl-coenzyme A dehydrogenase (Hadh) and Hadha decreased compared to the standard diet. In females, only Acadl increased in both 45% and 60% HFDs.

#### 2.2.5. Mitochondrial Respiratory Chain

**Figure 7:**
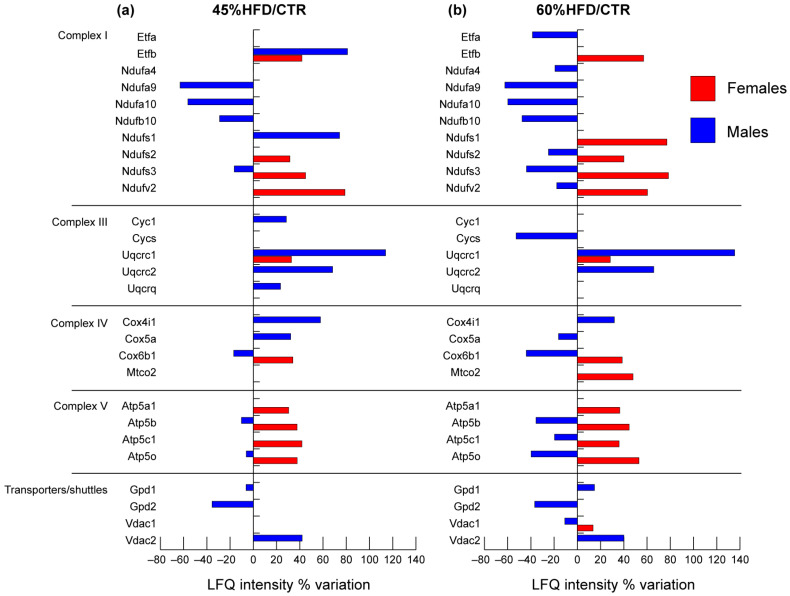
Proteomic analysis of mitochondrial respiratory chain proteins. Histograms of differentially expressed proteins (LFQ intensity% variation, ANOVA and Tukey’s test, n = 10, *p*-value < 0.05) in (**a**) 45% HFD vs. standard diet (CTR) female (red bars) and male (blue bars) mice; (**b**) 60% HFD vs. CTR female and male mice.

Concerning the mitochondrial respiratory chain, protein variations were different in males compared to females (Figure 7). In the latter, a high increase of all significant proteins was observed in both 45% and 60% HFD compared to CTR. In males, complex I components NADH dehydrogenase [ubiquinone] 1 alpha subcomplex subunits 9 and 10, beta subcomplex subunit 10 and iron-sulfur protein 3 (Ndufa9, Ndufa10, Ndufb10, Ndufs3) were decreased in both HFDs compared to control except for NADH-ubiquinone oxidoreductase subunit 1 (Ndufs1) and electron transferring flavoprotein B (Etfb) that increased in 45% HFD vs. control. The mitochondrial complex III components cytochrome b-c1 complex subunits 1, 2 and 8 (Uqcrc1, Uqcrc2, Uqcrq) and cytochrome c1 (Cyc1) were positively regulated by high-fat feeding, while complex V components ATP synthase subunit alpha, beta, gamma and O (Atp5a1, Atp5b, Atp5c1, Atp5o) were negatively affected. The component of complex IV cytochrome c oxidase subunit 5a (Cox5a) increased in 45% and decreased in 60% HFD. Cytochrome c oxidase subunit 6B1 (Cox6b1) was decreased, whereas cytochrome c oxidase subunit I (Cox4i1) increased in both 45% and 60% HFD vs. CTR.

Transporters of the inner and outer mitochondrial membranes were affected by high-fat feeding in both males and females. Voltage-dependent anion-selective channel protein 1 (Vdac1) increased in 60% HFD compared to control in females, while it showed an opposite trend in males. In the latter, an increase of voltage-dependent anion-selective channel protein 2 (Vdac2) was observed in both 45% and 60% HFD vs. CTR. Glycerol shuttle was negatively affected by diets since cytoplasmic (Gpd1) and mitochondrial (Gpd2) glycerol-3-phosphate dehydrogenases were decreased in 45% HFD vs. CTR. Gpd1 increased in 60% HFD.

### 2.3. Bioinformatics Analysis of Proteomics Results

Results from label-free proteomics analysis have been analyzed utilizing IPA software to predict biochemical pathways and functional biological processes associated with differentially expressed proteins. In particular, datasets of changed proteins in 45% HFD vs. CTR (i.e., 117 proteins for males and 33 for females) and in 60% HFD vs. CTR (i.e., 131 proteins for males and 41 for females) were analyzed. In the first step, a core analysis, starting from the list of changed proteins with their *p*-values and fold changes, was performed. This analysis allowed us to identify canonical pathways, downstream effectors, and upstream regulators predicted to be involved in response to high-fat intake onto skeletal muscle.

#### 2.3.1. Canonical Pathways

The canonical pathways analysis led to recognizing the key signaling pathways in which the differentially expressed proteins could be involved. A total of 9 pathways significantly associated with 45% HFD and 3 pathways associated with 60% HFD (Fisher’s right-tailed exact test *p*-value < 0.05 and z-score > 2 or <−2) were identified.

As reported in Table 1, in 45% HFD females compared to CTR, the main pathways highlighted by IPA software as activated were oxidative phosphorylation and TCA cycle. In 45% HFD males, the TCA cycle was activated, while oxidative phosphorylation was unchanged. Moreover, actin cytoskeleton signaling, regulation of actin-based motility by Rho, estrogen receptor signaling, gluconeogenesis and ILK signaling were activated in males, whereas RhoGDI and protein kinase A signaling was inhibited. In 60% HFD females, prediction remained unchanged compared to 45% HFD, whereas in males, TCA cycle activation was reduced, and oxidative phosphorylation was strongly inhibited.

#### 2.3.2. Diseases and Biofunctions

IPA was used to predict biological functional processes and disorders associated with differentially expressed proteins (Table 2). In 45% HFD females vs. CTR, predicted decreased functions, based on the z-score, were muscle necrosis and cell death. In contrast, in 45% HFD males compared to controls, a decrease in the transmembrane potential of mitochondria and activation of damage of muscle process together with an increment of carbohydrates, glycogen and fatty acids concentration were highlighted. 60% HFD female mice did not show significant changes, while in 60% HFD males vs. CTR, an increase of lipids and fatty acids concentration and a decrease of oxygen consumption and of transmembrane potential were predicted.

#### 2.3.3. Upstream Regulators

Upstream regulators analysis performed by IPA software allows identifying upstream factors predicted to regulate groups of proteins changed in our analysis. Table 3 represents upstream regulators predicted to be involved in 45% and 60% HFD females and males, respectively. Among the top upstream regulators controlling the expression of proteins changed in 45% HFD mice, insulin receptor (Insr), insulin-like growth factor 1 receptor (Igf1r), peroxisome proliferator-activated receptor gamma coactivator 1-alpha (Ppargc1a) and beta (Ppargc1b) and estrogen-related receptor alpha (Essra) were predicted to be activated. In contrast, carnitine palmitoyltransferase 1B (Cpt1b), caseinolytic mitochondrial matrix peptidase proteolytic subunit (Clpp), Rptor-independent companion of mTOR complex 2 (Rictor), tumor protein p53 (Tp53) and lysine demethylase 5A (Kdm5a) were predicted to be inhibited in females. The above-mentioned predicted regulators were also similarly expressed in 60% HFD females. In 45% HFD males, Myc proto-oncogene (Myc) and Insr were activated, while peroxisome proliferator-activated receptor gamma (Pparg), Clpp, and nuclear receptor subfamily 4 group a member 1 (Nr4a1) inhibited. Moreover, in 60% HFD male mice, Cpt1b, Rictor, regulatory associated protein of mTOR complex 1 (Rptor) and hypoxia-inducible factor 1 subunit alpha (Hif1a) were activated.

### 2.4. Validation of Ppargc1a and Pparg by Immunoblotting

**Figure 8:**
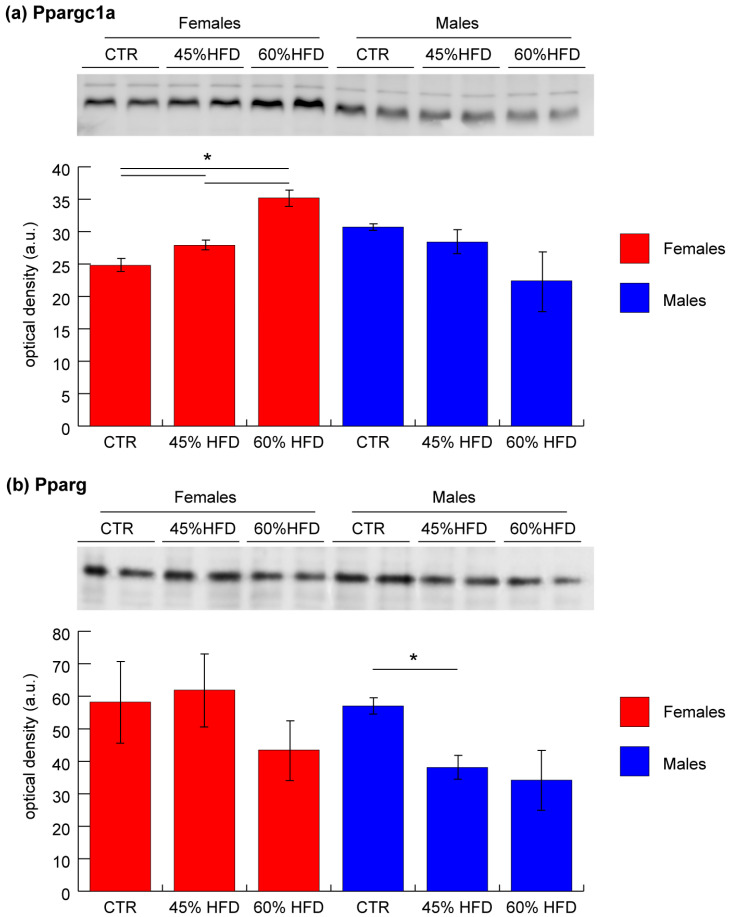
Representative histograms and immunoblot images of (**a**) peroxisome proliferator-activated receptor gamma coactivator 1-alpha (Ppargc1a) and (**b**) peroxisome proliferator-activated receptor gamma (Pparg) expression in gastrocnemius muscle (mean ± SD; * = significant difference, ANOVA and Tukey’s test, n = 2, *p*-value < 0.05) of CTR, 45% HFD and 60% HFD female (red) and male (blue) mice. Full-length images are available in Supplementary Figure S1.

To validate results from upstream regulator bioinformatic analysis, Ppargc1a and Pparg levels were assessed by immunoblotting. In female mice, in both 45% and 60% HFD compared to the control group, Ppargc1a was significantly increased (ANOVA and Tukey’s test, n = 2, *p*-value < 0.05), whereas in males, it was unchanged (Figure 8a). Pparg was significantly decreased in 45% HFD vs. CTR males, whereas it was unchanged in females (Figure 8b).

## 3. Discussion

This study aimed to characterize gender-related protein changes in animal models of diet-induced obesity (DIO). Results provided further evidence of the role of skeletal muscle in the molecular background of obesity and insulin resistance and contributed to identifying several regulatory molecules involved. The experimental setup included both male and female mice fed with two different high-fat diets (HFDs) containing 45% and 60% of fat compared to a standard diet (control group). A high-fat diet is extensively used to induce obesity in animal models and to investigate body modification associated with western-style diets. Continuous high caloric intake, particularly using food rich in saturated fatty acids, triggers metabolic disorders and insulin resistance that revealed several effects at multiorgan level [31]. Results obtained from model characterization showed that the HFD regimen increased body weight and insulin resistance in both male and female mice in a time and sex-dependent manner. The liver plays a major role in insulin resistance and hyperglycemia, concurring with elevated hepatic glucose production and impaired insulin-dependent suppression of glucose uptake during diabetes [32,33]. Increased hepatic lipid storage is one of the effects of insulin resistance [34]. We observed an increase of intrahepatic lipid content at 12 weeks with the highest dietary fat content for both sexes. In females, but not in males, this increase was also present in the 45% HFD despite the limited increment of weight. On the contrary, an increase of intra-abdominal fat was higher in males and already present within the 45% HFD. Taken together, data obtained from animal model characterization and MRI liver assessment confirmed that fat diets induce glucose intolerance and increase body weight, lipid content in liver and intra-abdominal fat, with several sex differences.

Many potential factors may contribute to gender differences in adipose tissue functions, including sex hormones, sex chromosomes, mitochondria, and epigenetic factors. Sex steroid hormones play a major role in sex differences in adipose tissue biology, distribution and inflammation. Studies show that a sex dimorphism exists in lipid storage with more fat accumulation in visceral adipose tissue (VAT) in men and more storage in subcutaneous adipose tissue (SAT) in women [35,36]. VAT and SAT are characterized by a different rate of lipolysis, which is higher in VAT; this can lead to the activation of proinflammatory chemokines and cytokines in males, whereas females are protected from inflammation due to their lower levels of lipolysis in obesity [37,38].

However, human and mouse male embryos are larger than female embryos even before the differentiation of the gonads, suggesting a role for sex chromosome dosage [39]. Interestingly, mouse models have highlighted the effects of sex chromosomes on the expression of hundreds of autosomal genes [40,41,42], including O-GlcNac transferase (OGT) and some of the nuclear-encoded mitochondrial genes. OGT mediates cell signaling through protein O-GlcNAcylation. Notably, OGT can participate in insulin resistance as a number of insulin signaling intermediates undergo O-GlcNAcylation [43]. OGT is also a transcriptional regulator that could control different expression networks and localizes to mitochondria, where it plays a role in regulating mitochondrial structure and functions [44].

Finally, gene expression can be regulated epigenetically. MicroRNAs (miRNAs) expression is known to be influenced by sex [45]. The X chromosome is enriched in miRNAs, which may escape X-linked inactivation [46]. miRNAs are reported to stimulate or inhibit the differentiation of adipocytes and to regulate specific metabolic and endocrine functions, and some of them with distinct sex-biased patterns in expression have been implicated in adipogenesis [47,48].

The above-mentioned factors are probably responsible for the differences in body weight gain observed with standard diet where CTR males still gained weight, while females not. This reflects a basal condition that is exacerbated by high-fat diets.

As concerns skeletal muscle, protein expression can be influenced by the same factors [49] and by differences in the levels of adipose tissue-derived circulating hormones, chemokines, cytokines, and inflammatory regulators [19].

At a molecular level, the skeletal muscles of males were more affected than females, particularly as concerned with the contractile and structural proteins. In males, extracellular matrix (ECM) and cytoskeleton dysregulation coupled with actin filaments and collagen deposition were observed. Increment of Col1a1 and a2 observed in 45% HFD mice suggests the onset of a connective tissue deposition, while the decrement of Col6a3 and Lum in both HFDs and Prelp, involved in ECM organization and anchoring to the basement membrane, in 60% HFD suggest impairment in muscle contraction. ECM remodeling and fibrosis are characteristic pathological features of obesity and have been observed in adipocytes and skeletal muscles [50]. ECM plays an important role in myocytes maintenance, myogenesis and regeneration and its homeostasis is granted by regulating catabolic and anabolic processes involving its components [50]. Among others, regulators of cell homeostasis are metalloproteinases (MMTs), inhibitors of MMTs and proteoglycans. Alterations of these factors lead to ECM overproduction (fibrosis) and reduction of the muscle contractile force. Our results indicate that lumican decreased in mice after feeding with 45% and 60% HFDs. Fibrosis in diet-induced obesity results from intra-myocytes lipid deposition and low-grade chronic inflammation, which impair muscle repair and regeneration by interfering with the recruitment and activity of macrophages, satellite cells and fibroblasts [51]. Furthermore, the loss of actin-associated proteins like Cfl2, Capza2 and Tmod4 suggests modifications of cytoskeletal architecture resulting in actin-filament accumulation. In cytoskeleton, actin capping proteins, under physiological conditions, prevent actin depolymerization and regulate actin organization. The increased expression of beta-actin is in line with previous considerations, supporting the hypothesis of actin filaments accumulation. Overall, the increment of Col1a1, Col1a2 and Actb and the decrement of Lum, Prelp, Col6a3, Cfl2, Capza2 and Tmod4 suggest that the skeletal muscle stiffness is increased at both cellular and ECM level in a diet-dependent mode.

As concerns, contractile proteins, a clear reduction in slow-type myosin fibers (Myh7, Myl2, Myl3) in association with increased fast-type (Myh2, Mylpf, Mybpc2, Myl1) in 60% HFD mice compared to controls was observed in male mice thick filaments. Conversely, females showed few alterations of contractile and structural proteins highlighting the absence of deleterious effects carried out in males by the high-fat diet. Slow-type muscle fibers are rich in mitochondria and are characterized by oxidative metabolism associated with high insulin sensitivity compared to fast-type muscle fibers, either oxidative or glycolytic [52]. In humans, a lower proportion of slow-type muscle fibers in patients with metabolic syndrome and obesity, inversely related to the degree of insulin resistance, was described [53,54,55,56]. In animal models, impairment of slow-twitch muscles (e.g., soleus) in association with insulin resistance was reported in mice fed with a long-term (12 weeks) HFD [57]. The increment in fast-type fibers and a slow-type fibers reduction can be derived by Figure 4 and correlate with studies in humans and DIO animal models, further supporting the association between the muscle contractile machinery, insulin resistance and obesity. The rearrangement in fiber type distribution impacts muscle metabolism because fast-type muscle fibers can produce energy also in the presence of mitochondrial dysfunction, oxidative stress and obesity-induced inflammation enhancing their glucose metabolism and reducing the efficiency of oxidative phosphorylation [52].

Noteworthy, metabolic alterations were more evident in males than females and were confirmed by bioinformatic analyses (Table 1 and Table 2). In particular, the former showed enhanced anaerobic metabolism, oxidative stress and mitochondrial dysfunction in response to both 45% and 60% high-fat feeding. In 60% HFD male mice, glycolysis was active; however, the level of Ldha was increased, suggesting a high production of lactate from pyruvate, which is a distinctive trait of anaerobiosis. This phenomenon can be explained by the reduced level of mitochondrial oxidative phosphorylation due to a low-chronic inflammation, oxidative stress and mitochondrial dysfunction typical of obese mice. Furthermore, glycogenolysis was enhanced, suggesting that glycogen can become a source for glucose due to a reduced insulin-dependent glucose uptake typical of HFD-induced insulin resistance. Results from J. Lovejoy et al. and J. E. Friedman et al. indicated an association between insulin resistance and elevated basal lactate levels in obese subjects [58], as for human skeletal muscle cells [59]. At variance, only two proteins (Gys1 and Eno1) were changed in gastrocnemius muscles of females suggesting that changes described for males are absent in females under HFD.

At mitochondrial level, the effects of high-fat feeding on the TCA cycle and beta-oxidation were present both in female and male mice. The latter, fed with 45% HFD, showed an increase of enzyme levels of the beta-oxidation pathway suggesting that FFAs are degraded under this condition. Conversely, when the level of fats increased (60% HFD), a partial reduction of oxidative enzymes was observed. This phenomenon can be associated with oxidative stress onset and mitochondrial dysfunction with the inability of cells to compensate for the increase of FFAs intake. Moreover, in the skeletal muscle of extremely obese subjects, a reduction in the FFAs oxidation has been described [60,61]. Similar behavior is observed for proteins of the TCA cycle, which increased in males fed with 45% HFD and partially decreased in males fed with 60% HFD. It can be concluded that the consequences of high-fat feeding on beta-oxidation and TCA cycle in males are fat-content dependent. Concerning female mice, beta-oxidation was unaltered, while enzymes of the TCA cycle were increased at both 45% and 60% of fat content. These results underline the absence of mitochondrial damage and oxidative stress and the capacity to utilize the excess of FFAs as an energy source. It has been observed that in the skeletal muscle of subjects affected by insulin resistance, the switch between glucose to fat oxidation is impaired compared to healthy subjects [62]. We can postulate from these results that a different response to HFD at muscle level in males and females can be related to the absence of insulin resistance in the latter.

Collectively, variations from our study indicate alteration of proteins of the mitochondrial respiratory chain in all complexes, except for complex II, in both males and females. In females, the respiratory chain was upregulated under both high-fat feedings indicating an efficient metabolic adaptation allowing the consumption of a higher amount of FFAs as an energetic source counteracting the mitochondrial damages. Conversely, males exhibited a decrement of complex I, IV and V at a lower extent in 45% HFD vs. CTR and at a higher extent in 60% HFD vs. CTR according to microarray and real-time quantitative PCR results that indicate HFD as an important factor in downregulation of gene expression of mitochondrial oxidative phosphorylation in insulin-resistant humans and animal models [63]. Furthermore, complex III was increased in both 45% HFD and 60% HFD compared to control. Complex III is the main producer of reactive oxygen species (ROS) within the mitochondrial respiratory chain [64]. Thus, increased expression of complex III subunits can be a signal for mitochondrial oxidative stress.

Damages related to oxidative stress were exacerbated by the reduction of enzymes involved in oxidative stress protection (see supplementary Figure S2). The overall enzyme reduction in male mice fed with HFD suggests the presence of oxidative stress, which is in line with metabolic alterations observed in mitochondria [65,66,67]. This metabolic profile suggests the possible development of insulin resistance in skeletal muscle of male mice fed with HFD. Females did not show this trait and were characterized by the absence of metabolic alteration, mitochondrial dysfunction and oxidative stress, even under high-fat intake.

Regarding transport proteins (see Supplementary Figure S3), changes can be appreciated concerning the decrement of FFAs transport proteins in males under 60% high-fat feeding, highlighting the absence of transport of fatty acids even in the presence of higher FFAs intake. This can be associated with the inability of males to use the excess of FFAs as an energy source. Females were characterized by an opposite trend.

Upstream regulators analysis (Table 3) indicated the presence of factors potentially involved in the modulation of altered pathways in our analysis: besides the activation of Insr, Igf1r and Essra, which are well known critical and protective factors in regulating metabolic homeostasis and insulin sensitivity [68,69], Ppargc1a and Ppargc1b were activated, while Cpt1b, Clpp, Tp53 and Kdm5a inhibited in females under both 45% and 60% HFDs, contributing to improving glucose tolerance and counteracting insulin resistance. Ppargc1a and its structural homolog Ppargc1b are transcriptional coactivators involved in regulating energy metabolism in response to different stimuli [70,71,72,73,74]. It has been described that increased expression of Ppargc1a in muscle cells allows the recovery of levels of insulin-sensitive glucose transporter 4 (Glut4) by coordinating the transcription factor Mef2c [73,75]. Validation of Ppargc1a by immunoblotting has been performed, confirming the increased levels in both 45% and 60% HFD females vs. CTR and a tendential (not supported by statistical analysis) decrease in males. Clpp is a mammalian quality control protease that plays an important role in initiating the mitochondrial unfolded protein response. Knockout of Clpp in mice increased whole-body energy expenditure and mitochondrial biogenesis. When challenged with a high-fat diet, despite similar caloric intake, knockout mice are protected from diet-induced obesity, glucose intolerance and insulin resistance [76,77]. Cpt1b is the rate-limiting enzyme governing long-chain fatty acid entry into mitochondria [78]. Concerning Tp53, its role in regulating mitochondrial function and oxidative stress is well documented [79]. In rats that develop early insulin resistance and T2DM, exercise training decreased p53 protein levels and restored mitochondrial function in skeletal muscle [80]. These findings suggested that p53 may enhance developing insulin resistance by inducing oxidative stress in skeletal muscle [81]. Kdm5a is instead a repressor of genes controlling mitochondrial function and differentiation [82].

Notably, Insr and Clpp had the same expression in 45% HFD male mice, but not under 60% HFD. Furthermore, in 45% HFD males, their protective effects were challenged by the inhibition of Pparg and Nr4a1 signaling [83,84]. Validation of Pparg by immunoblotting confirmed the decreased levels in 45% HFD and a tendential (not supported by statistical analysis) decrease in 60% HFD males vs. CTR, whereas in females, it was unchanged.

In 60% HFD male mice, the picture is worsened by the activation of Cpt1b together with Rptor, Rictor, and Hif1a. Rptor and Rictor are two components of mTOR complexes, respectively mTORC1 and mTORC2. The mTOR pathway is thought to be an important regulator of insulin action on glucose metabolism. It has been described that the mTOR pathway is overactivated in the liver and muscle of obese high-fat-fed rats [85]. Hif1a, a master transcription factor of oxygen homeostasis, induces inflammation and insulin resistance in obesity, targeting mainly adipose tissue [86]. In muscle, the activation of Hif1a signaling causes inactivation of the TCA cycle and oxidative phosphorylation with the consequent lowering of aerobic capacity, increasing the risk for diet-induced insulin resistance [87].

## 4. Materials and Methods

### 4.1. Animals, Diet Administration and Glucose Tolerance Test

The study was performed on 15 C56BL/J female and 15 male mice. Animals were maintained and handled in compliance with institutional guidelines for the care and use of experimental animals and national laws for animals used in research (Prot. N. 6B2B3.44 D.lsg. 116/1992 and N. 29/2018-PR D.lsg. 26/2014). Mice were housed in the San Raffaele Hospital animal facility with access ad libitum to food and water and maintained in a 12/12 h light/dark cycle. At 5 weeks of age, animals were divided into three groups (5 female and 5 male mice each group) and fed with different diets for 14 weeks: a standard diet (10 kcal% fat) (control group, CTR), a 45 kcal% fat content diet (45% high-fat diet, HFD) and a 60 kcal% fat content diet (60% HFD). Mice were constantly monitored for their body weight and blood glucose level. Glucose tolerance test (GTT) was performed during fasting condition at −4 and 7 weeks from the diet, and blood was collected from the tail using a glucometer (StatStrip Xpress^®^2, Nova Biomedical, MA, USA) after i.p. glucose injection (2 g/kg of bw, dissolved in saline) at 0, 15, 30, 45, 60 and 120 min.

### 4.2. MRI Study

Magnetic resonance imaging (MRI) was performed on 4 male and 4 female mice in each group and completed two-time point of analysis, an early time point at 4 weeks and a late one at 12 weeks of diet, based on the literature [88].

All MRI studies were performed in a 7T preclinical scanner (BioSpec 70/30 USR, Paravision 6.0.1, Bruker BioSpin, Billerica, MA, USA), equipped with 450/675 mT/m gradients (slew-rate: 3400–4500 T/m/s; rise time 140 µs) and a circularly polarized mouse body volume coil with an inner diameter of 40 mm. All mice undergoing imaging were under gaseous anesthesia (Isoflurane, 3% for induction and 2% for maintenance in 2 L/min oxygen); lying prone on a dedicated temperature control apparatus to prevent hypothermia, with breathing rate and body temperature continuously monitored (SA Instruments, Inc., Stony Brook, NY, USA).

Axial 2D high-resolution (HR) rapid acquisition with relaxation enhancement (RARE) T2 images were obtained. Images with (FS) or without fat suppression (NFS) (repetition time (TS) = 3000 ms; echo time (TE) = 40ms; rare factors = 8: field of view (FOV) = 230 × 20 mm; matrix = 224 × 192 (resolution= 0.143 × 0.104)) were acquired to measure liver and intra-abdominal adipose tissue volumes. Enough slices were acquired to allow imaging of the liver and kidney region. Liver volume was calculated by the manual contour of the RARE T2 sequence. Axial RARE T2 images with FS and NFS were used to measure intra-abdominal fat volume using MIPAV 8.0.2 (National Institutes of Health, Bethesda, MA, USA). Three adjacent slices per animal were selected from a slice in which renal pelvises had approximately the same dimensions, both in FS and NFS images. Segmentation was performed by subtracting FS from NFS images. Multispectral fuzzy C-means clustering algorithm was applied on the resulting whole images to perform “hard and fuzzy segmentation”. Fat tissue volume quantification was finally calculated using the Paint Grow tool on each slice.

Proton magnetic resonance spectroscopy (1H-MRS) was performed to extract the hepatic lipid content (HLC). Multi-slice gradient-echo images (TR = 3000 ms, echos = 20, TE = from 6.78 to 135.63 ms, matrix =128 × 128 (resolution = 0.250 × 0.156 mm), FOV = 320 × 20 mm) were used for liver identification and definition of a (3 × 3 × 3) mm^3^ volume of interest for 1H-MRS. MR spectra were acquired with point-resolved spectroscopy (PRESS) technique (TS = 2500MS, TE = 16.6, spectral width = 4504.50 Hz), reconstructed with Topspin program (PV6.0, Bruker BioSpin, Billerica, MA, USA) and analyzed using Mnova program (Mestrelab Research S.L., Santiago de Compostela, A Coruña, Spain). The integral of each peak was determined in water suppression spectra to extract HLC, as previously described [88].

### 4.3. Statistical Analysis

Statistical analysis of diet effect evaluation (body weight, GTT and MRI analysis) was performed using Prism 7 (GraphPad Software Inc., San Diego, CA, USA). Two-way ANOVA was computed to investigate several variables (time × diet), followed by Tukey’s post hoc test for multiple comparisons. A statistically significant difference was accepted when *p*-value ≤ 0.05.

### 4.4. Muscle Biopsies, Protein Extraction and Quantification

At the end of the 14 week diet, mice were euthanized by general anesthesia (4% isoflurane in the air), and gastrocnemius muscles were collected from each animal and stored at −80 °C. For label-free proteomics analysis, an aliquot of each frozen muscle was suspended in lysis buffer (urea 7 M, thiourea 2 M, 3-[(3-Cholamidopropyl)dimethylammonio]-1-propanesulfonate hydrate, CHAPS 4%, Tris 30 mM and phenylmethylsulfonyl fluoride, PMSF 1 Mm) and solubilized by sonication on ice. Protein concentration in each sample was determined by 2D Quant Kit (GE Healthcare, Little Chalfont, Buckinghamshire, UK). For each sample, proteins (100 µg) were selectively precipitated using a PlusOne 2D-Clean-up kit to remove nonprotein impurities and resuspended in 50 mM ammonium bicarbonate and 0.1% RapiGest SF surfactant (Waters, Milford, MA, USA). Quantification with Pierce bicinchoninic acid (BCA) protein assay (Thermo Fisher Scientific, Rodano, Italy) was then performed.

### 4.5. Label-Free Liquid Chromatography with Tandem Mass Spectrometry (LC–MS/MS)

Protein extracts were reduced for 45 min in 5 mM dithiothreitol (DTT) at 60 °C, carbamidomethylated for 45 min in 15 mM iodoacetamide, and digested with sequence grade trypsin (Promega, Madison, WI, USA) for 16 h at 37 °C using a protein:trypsin ratio of 50:1. After acidification with trifluoracetic acid and desalting on C18 tips (Zip-Tip C18 micro, Merck Millipore, Milano, Italy), peptide samples were vacuum concentrated, reconstituted in HPLC buffer A (0.1% formic acid) and separated on a Dionex UltiMate 3000 HPLC System with an Easy Spray PepMap RSLC C18 column (250 mm, internal diameter of 75 μm) (Thermo Fisher Scientific Rodano, Italy), adopting a five steps acetonitrile (ACN)/formic acid gradient (5% ACN in 0.1% formic acid for 5 min, 5–35% ACN in 0.1% formic acid for 139 min, 35–60% ACN in 0.1% formic for 40 min, 60–100% ACN for 1 min, 100% ACN for 10 min, at a flow rate of 0.3 μL/min), and electrosprayed into an Orbitrap Tribrid Fusion (Thermo Fisher Scientific Rodano, Italy). The LTQ-Orbitrap was operated in a positive mode in data-dependent acquisition mode to automatically alternate between a full scan (350–2000 m/z) in the Orbitrap (at resolution 60000, AGC target 1000000) and subsequent CID MS/MS in the linear ion trap of the 20 most intense peaks from full scan (normalized collision energy of 35%, 10 ms activation). Isolation window: 3 Da, unassigned charge states: rejected, charge state 1: rejected, charge states 2+, 3+, 4+: not rejected; dynamic exclusion enabled (60 s, exclusion list size: 200). Mass spectra were analyzed using MaxQuant software (Max-Planck-Institute of Biochemistry, Munich, Germany, version 1.6.3.3). The initial maximum allowed mass deviation was set to 6 ppm for monoisotopic precursor ions and 0.5 Da for MS/MS peaks. Enzyme specificity was set to trypsin/P, and a maximum of two missed cleavages was allowed. Carbamidomethylation was set as a fixed modification, while N-terminal acetylation and methionine oxidation were set as variable modifications. The spectra were searched by the Andromeda search engine against the *Mus musculus* Uniprot UP000000589 sequence database (55,474 proteins, release 18 October 2020). Protein identification required at least one unique or razor peptide per protein group. Quantification in MaxQuant was performed using the built-in extracted ion chromatogram (XIC)-based label-free quantification (LFQ) algorithm using fast LFQ. The required FDR was set to 1% at the peptide, 1% at the protein and 1% at the site-modification level, and the minimum required peptide length was set to 7 amino acids. Statistical analyses were performed using the Perseus software (Max Planck Institute of Biochemistry, Munich, Germany, version 1.4.0.6). Each sample was run in duplicate. For each experimental group, the proteins identified in at least 80% of samples were considered. For statistical analysis, ANOVA test and multiple samples tests with a *p*-value threshold of 0.05 were applied, and results revealed the variation of protein expression between the control group (standard diet), 45% HFD-fed mice, and 60% HFD-fed mice. To exclude the presence of false positives from the analysis, Benjamini–Hochberg false discovery rate test was applied. To facilitate the examination of results, varied proteins were divided into functional groups.

### 4.6. Ingenuity Pathway Analysis

Functional and network analyses of statistically significant protein expression changes were performed through Ingenuity Pathway Analysis (IPA) software (Qiagen, Hilden, Germany). In brief, data sets with protein identifiers, statistical test *p*-values and fold change values calculated from the mass-spectrometry experiment were analyzed by IPA. The “core analysis” function was used to interpret the data through the analysis of biological processes, canonical pathways, upstream transcriptional regulators enriched with differentially regulated proteins. Then the “comparison analysis” function was used to visualize and identify significant proteins or regulators across experimental conditions. *P*-values were calculated using a right-tailed Fisher’s exact test. Activation z-score was used to predict the activation/inhibition of a pathway/function/regulator [89]. A Fisher’s exact test *p*-value < 0.05 and a z-score < −2 and >2, which takes into account the directionality of the effect observed, were considered statistically significant.

### 4.7. Immunoblotting

Protein extracts (50 μg) from pooled CTR, 45% and 60% HFD, female and male gastrocnemius muscle samples were loaded in duplicate and resolved on 12% gradient polyacrylamide gels. Blots were incubated with rabbit polyclonal anti-Ppargc1a (Santa Cruz Biotechnology, Dallas, TX, USA, sc-13067, 1:500) and anti-Pparg (Santa Cruz Biotechnology, sc-6285, 1:500). After washing, membranes were incubated with anti-rabbit (GE Healthcare, 1:10,000) or anti-goat (Santa Cruz Biotechnology, 1:5000) secondary antibody conjugated with horseradish peroxidase. Signals were visualized by chemiluminescence using the ECL Prime detection kit and the Image Quant LAS 4000 (GE Healthcare) analysis system. Band quantification was performed using the Image Quant TL (GE Healthcare) software followed by statistical analysis (ANOVA + Tukey, n = 2, *p*-value < 0.05). Band intensities were normalized against the total amount of proteins stained by Sypro ruby total-protein stain.

## 5. Conclusions

Overall, our study clearly showed that HFD induces weight gain and systemic modification resembling metabolic syndrome. These effects were particularly evident in males and in animals fed with the highest fat regimen. Moreover, our results on liver and muscle clearly underline the presence of a gender-specific response that should be considered when approaching obesity and related comorbidities.

Proteomic and bioinformatic analyses of skeletal muscle confirmed gender differences in DIO animal models fed for 14 weeks with 45% or 60% HFD vs. standard diet. Female mice showed only a few alterations of contractile and structural proteins in response to both HFDs and were characterized by unaltered glucose metabolism coupled with unchanged glycogenolysis and lactate production, indicating that the aerobic metabolism was active. Additionally, beta-oxidation and TCA cycle enzymes expression was increased, indicating the ability of females to utilize the excess fat intake as a metabolic energy source. Furthermore, mitochondrial respiratory chain complexes were maintained, suggesting the absence of mitochondrial dysfunctions.

At variance, male mice showed ECM and cytoskeleton protein dysregulation coupled with actin filaments and collagen deposition, which indicate an increased muscular stiffness at both ECM and cellular levels. Fiber type switch from oxidative/slow to glycolytic/fast was observed in 60% HFD suggesting a predisposition for the insulin resistance onset. Metabolic alterations worsened passing from 45% to 60% HFD and involved anaerobic metabolism, oxidative stress and mitochondrial dysfunction. Glycolysis was active, but the level of activity of Ldha was increased, indicating the onset of anaerobic metabolism. Furthermore, glycogenolysis was enhanced, suggesting glycogen as the main source of glucose in response to low levels of insulin-mediated glucose uptake. At mitochondrial level, TCA cycle, FFAs beta-oxidation and respiratory chain complex I, IV and V were decreased, while complex III, which is involved in ROS production, increased, supporting developing oxidative stress. Damages related to ROS production were exacerbated by the reduction of enzymes involved in oxidative stress protection. This metabolic profile suggests the possible development of insulin resistance in skeletal muscle of male mice fed with HFD and suggests the onset of sarcopenic obesity. To elucidate this point, further analyses involving mice fed for a longer time with the same diets will be carried out in our laboratory.

Pathways analysis of upstream regulators indicated the presence of factors potentially involved in the modulation of pathways resulted altered in our analysis. Due to their regulatory role, the level of expression of these factors in response to the HFDs would contribute to better characterize this condition and to find potential candidates as therapeutic targets or biomarkers.

## Figures and Tables

**Table 1:** Canonical pathways heat map displaying the most significant results (ordered by decreasing z-scores in females, then in males) across datasets in 45% HFD vs. control (left panel) and in 60% HFD vs. control (right panel). The orange and blue-colored rectangles indicate predicted pathway activation or predicted inhibition, respectively, via the z-score statistic. Pathways in common between the diet regimens are shown in italics.

Canonical Pathways 45% HFD/CTR	Females	Males	Canonical Pathways 60% HFD/CTR	Females	Males
*Oxidative phosphorylation*	3	0	*Oxidative phosphorylation*	3.162	−2.5
*TCA cycle II (eukaryotic)*	2.236	2.646	*TCA cycle II (eukaryotic)*	2.449	0.378
Actin cytoskeleton-signaling	N/A	3.207	Glycolysis I	N/A	2.121
Estrogen-receptor-signaling	N/A	2.449			
Regulation of actin-based motility by Rho	N/A	2.236			
Gluconeogenesis I	N/A	2.236			
ILK-signaling	N/A	2.138			
Protein kinase A-signaling	N/A	−2.236			
RhoGDI-signaling	N/A	−2.449			

Italics identifies items in common between diet regimens.

**Table 2:** Diseases and biofunctions heat map displaying the most significant results (ordered by decreasing z-scores in females, then in males) across datasets in 45% HFD vs. control (left panel) and in 60% HFD vs. control (right panel). The orange and blue-colored rectangles indicate predicted pathway activation or inhibition, respectively, via the z-score statistic. Functions in common between the diet regimens are shown in italics.

Diseases and Bio Functions 45% HFD/CTR	Females	Males	Diseases and Bio Functions 60% HFD/CTR	Females	Males
Cell death of muscle cells	−2	0.302	Concentration of lipid	N/A	2.779
Necrosis of muscle	−2.236	N/A	*Concentration of fatty acid*	N/A	1.99
Necrosis	−2.927	0.154	*Transmembrane potential of mitochondria*	N/A	−1.964
Damage of muscle	N/A	1.982	Consumption of oxygen	N/A	−1.982
Quantity of glycogen	N/A	1.977			
Quantity of carbohydrate	N/A	1.821			
*Concentration of fatty acid*	N/A	1.65			
Cellular homeostasis	N/A	−1.644			
Transmembrane potential	N/A	−2.2			
*Transmembrane potential of mitochondria*	N/A	−2.2			

Italics identifies items in common between diet regimens.

**Table 3:** Upstream regulators heat map displaying the most significant results (ordered by decreasing z-scores in females, then in males) across datasets in 45% HFD vs. control (left panel) and in 60% HFD vs. control (right panel). The orange and blue-colored rectangles indicate predicted regulator activation or inhibition, respectively, via the z-score statistic. Regulators in common between the diet regimens are shown in italics.

Upstream Regulators 45% HFD/CTR	Females	Males	Upstream Regulators 60% HFD/CTR	Females	Males
*Insr*	3.561	2.02	*Insr*	3.38	0.191
*Ppargc1a*	3.124	0.904	*Ppargc1a*	3.279	−0.299
*Rb1*	2.449	0.335	*Rb1*	2.333	−0.709
*Igf1r*	2.236	0.632	*Igf1r*	2.236	−0.905
Esrra	2.219	0.154	Pten	2.182	0.688
*Ppargc1b*	2	1.026	Stk11	2.121	0.471
Myc	0.831	2.213	*Ppargc1b*	2	−0.396
Nr4a1	−1	−3.174	Map4k4	−2.236	0.535
*Tp53*	−2.111	−1.035	*Kdm5a*	−2.333	0.816
*Kdm5a*	−2.449	−0.626	*Tp53*	−2.887	−0.671
*Rictor*	−3	−0.258	*Rictor*	−3.162	2
*Clpp*	−3.051	−2.449	*Clpp*	−3.207	0.209
*Cpt1b*	−3.138	−0.249	*Cpt1b*	−3.293	2.467
Pparg	N/A	−2.18	Hif1a	N/A	2.211
			Rptor	N/A	2.425

Italics identifies items in common between diet regimens.

## Data Availability

Data supporting reported results can be found in Supplementary Material Tables S1 and S2. Data from this study other than those published in this work are under privacy regulations but can be obtained on a case-to-case basis upon reasonable request to the corresponding author.

## Supplementary Materials

The following are available online at https://www.mdpi.com/article/10.3390/ijms22094680/s1, Table S1: list of changed proteins in male mice by label-free LC–ESI–MS/MS analysis, Table S2: list of changed proteins in female mice by label-free LC–ESI–MS/MS analysis, Figure S1: full-length immunoblot images, Figure S2: histograms of differentially expressed stress response proteins, Figure S3: histograms of differentially expressed transport proteins.
