# Supplementary material for: Skeletal Muscle Proteomic Profile Revealed Gender-Related Metabolic Responses in a Diet-Induced Obesity Animal Model
Source: Int J Mol Sci. 2021 Apr 28;22(9):4680. doi: 10.3390/ijms22094680 (PMC8125379; doi:10.3390/ijms22094680)
Supplement: Supplementary file 1 [file ijms-22-04680-s001.zip › AMANDA Supplementary material/Supplementary Figures S1 S2 S3.docx]

**Ppargc1a**


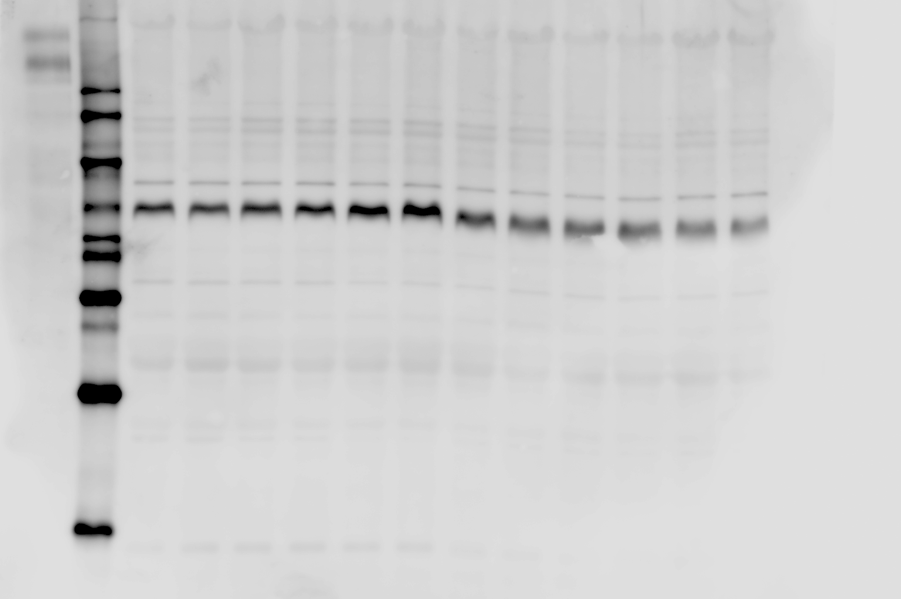


KDa

80

60

50

40

30

**Pparg**


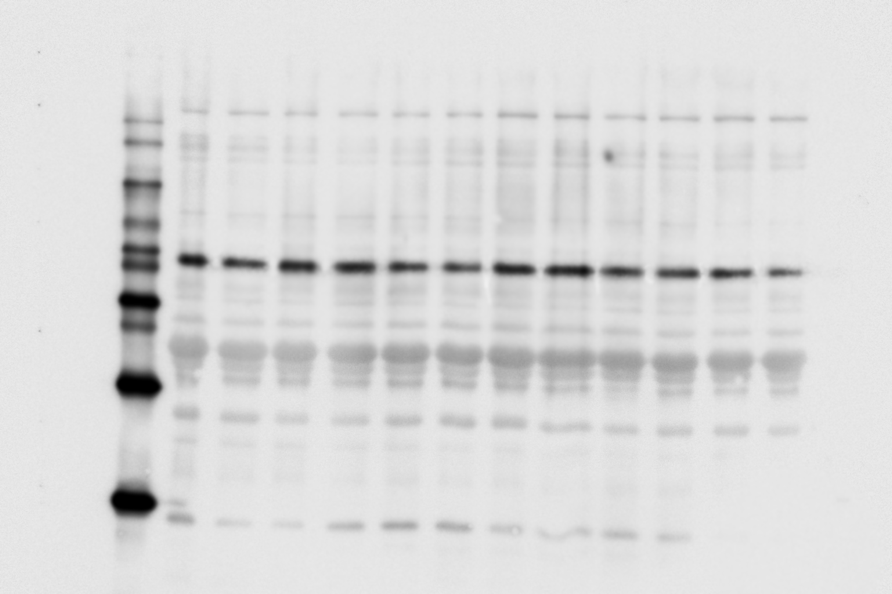


KDa

80

60

50

40

30

**Total stain**


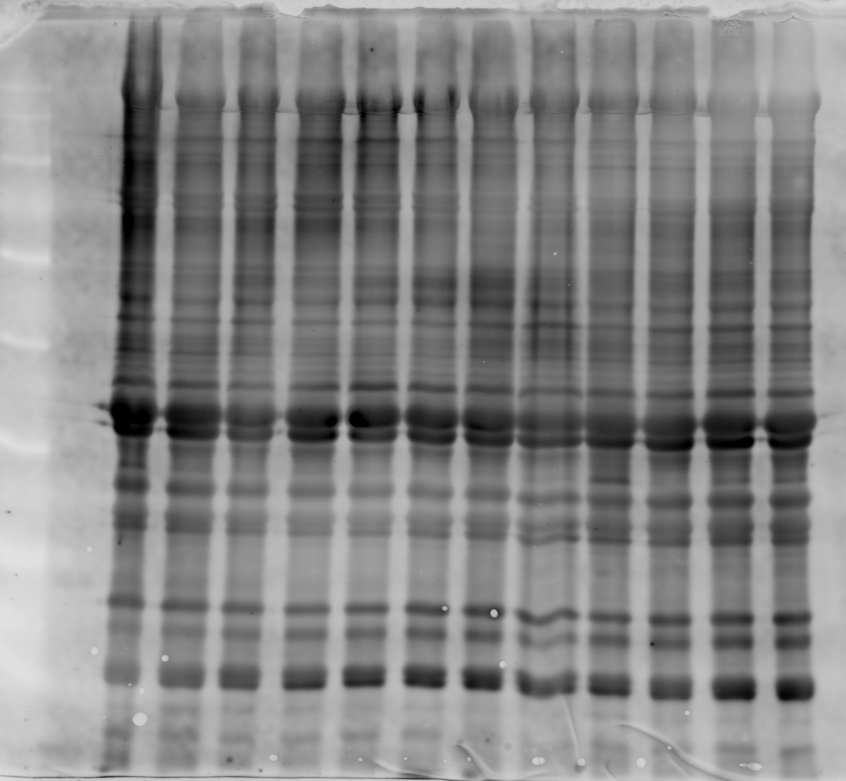


**Supplementary Figure S1. Full-length immunoblot images.** Protein extracts (50 μg) from pooled CTR, 45% and 60 % HFD, female and male gastrocnemius muscle samples were loaded in duplicate and resolved on 12% gradient polyacrylamide gels. Band intensities were normalized against the total amount of proteins stained by Sypro ruby total-protein stain.


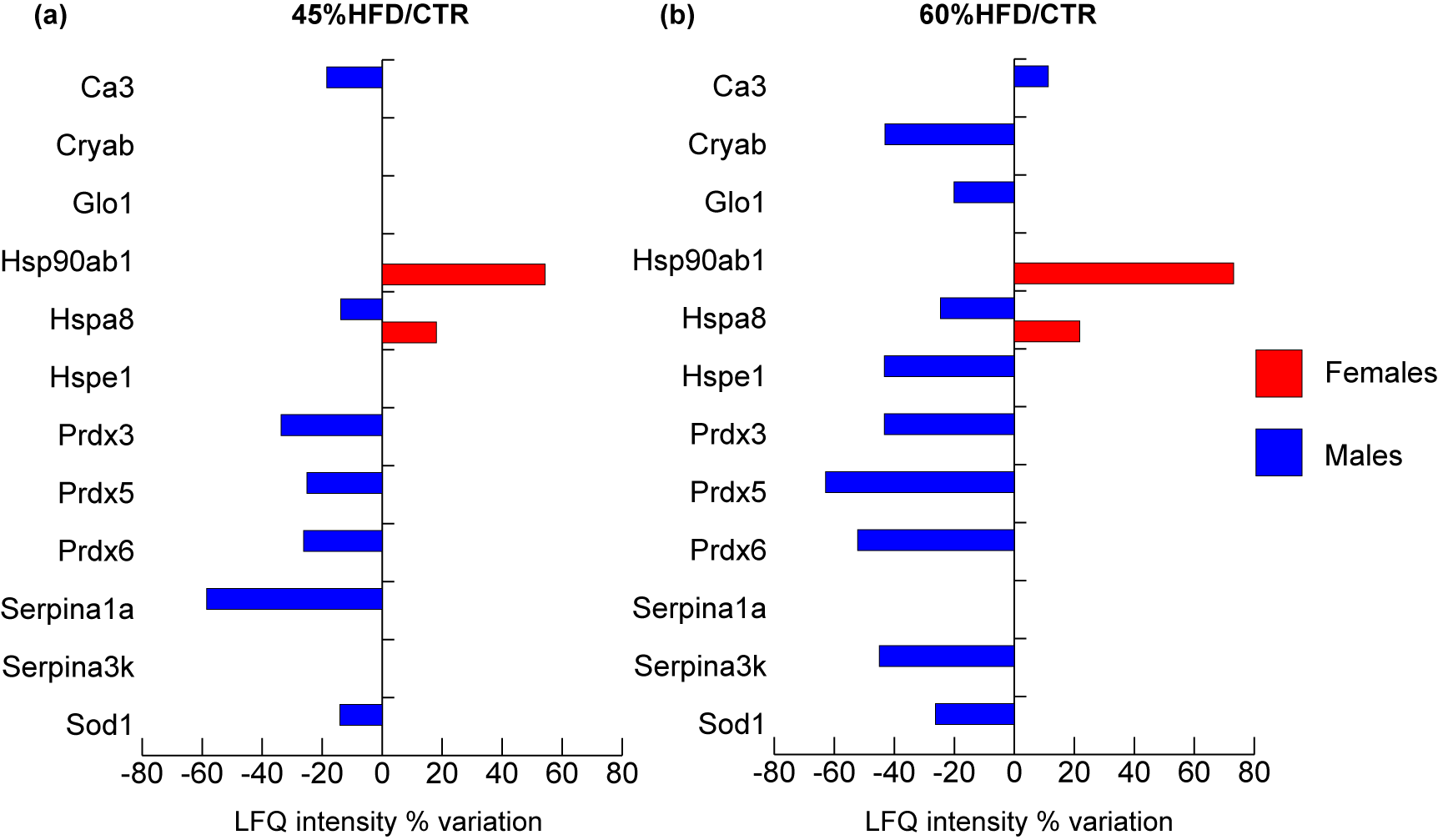


**Supplementary Figure S2.** **Proteomic analysis of stress response proteins.** Histograms of differentially expressed proteins (Label-free quantification, LFQ intensity % variation, ANOVA and Tukey’s test, n = 10, p < 0.05) in (a) 45% HFD vs. standard diet (CTR) female (red bars) and male (blue bars) mice; (b) 60% HFD vs. CTR female and male mice.

Several proteins involved in cellular stress response were at variance in our study. Lactoylglutathione lyase (Glo1), alpha-crystallin B chain (Cryab), 10 kDa heat shock protein (Hspe1) and serine protease inhibitor A3K (Serpina3k) were unchanged in 45% HFD compared to standard diet while thioredoxin-dependent peroxide reductase (Prdx3), peroxiredoxin-5 (Prdx5), peroxiredoxin-6 (Prdx6), superoxide dismutase (Sod1), carbonic anhydrase 3 (Ca3), heat shock cognate 71 kDa protein (Hspa8), serine protease inhibitor 1-1 (Serpina1a) were decreased in male mice. In 60% HFD vs CTR all proteins were decreased with the exception of Ca3 and Serpina1a. Serpina1a was unchanged whereas Ca3 increased. In females, only Hspa8 and heat shock protein HSP 90-beta (Hsp90ab1) increased in both 45% and 60% HFD compared to CTR.


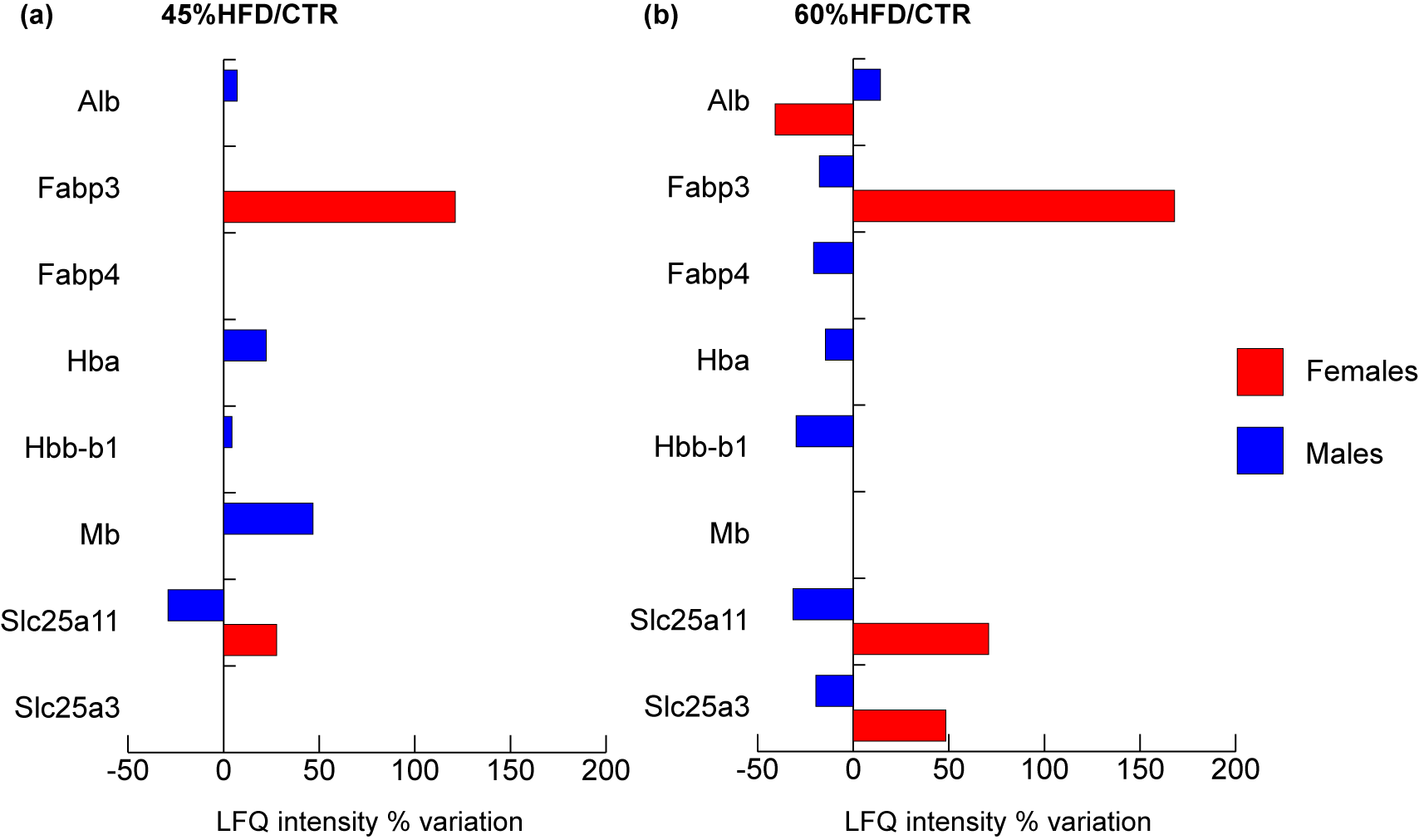


**Supplementary Figure S3.** **Proteomic analysis of transport proteins.** Histograms of differentially expressed proteins (LFQ intensity % variation, ANOVA and Tukey’s test, n = 10, p < 0.05) in (a) 45% HFD vs. standard diet (CTR) female (red bars) and male (blue bars) mice; (b) 60% HFD vs. CTR female and male mice.

Proteins involved in transport were at a variance in the two rodent’s groups. In males, beta-globin (Hbbt1), hemoglobin subunit alpha (Hba), myoglobin (Mb) and serum albumin (Alb) increased in 45% HFD compared to control, whereas a decrement of Hbbt1, Hba, fatty acid-binding protein 3 and 4 (Fabp3 and Fabp4) and increment of Alb were observed in 60% HFD compared to control. Females were characterized by increase of Fabp3 in 45% and 60% compared to CTR whereas Alb decreased in 60% HFD vs CTR only. Mitochondrial 2-oxoglutarate/malate carrier protein (Slc25a11) and phosphate carrier protein (Slc25a3) decreased in males and increased in females in both 45% and 60% HFD vs CTR.
